# Long-Term Outcomes of Endoscopic Transpapillary Gallbladder Stenting for Symptomatic Cholelithiasis

**DOI:** 10.1016/j.gastha.2026.101049

**Published:** 2026-07-03

**Authors:** Kazuki Natsui, Seiichi Yoshikawa, Kazuya Takahashi, Ayano Kagata, Kohtaro Yoshida, Naoya Fukushima, Natsuki Ishikawa, Yohei Koseki, Yui Natsui, Keisuke Morita, Yuki Nishigaki, Tsutomu Miura, Manabu Takeuchi, Shuji Terai

**Affiliations:** 1 Department of Gastroenterology, Nagaoka Red Cross Hospital, Nagaoka, Niigata, Japan; 2 Division of Gastroenterology and Hepatology, Graduate School of Medical and Dental Sciences, Niigata University, Chuo-Ku, Niigata, Japan

**Keywords:** Cholelithiasis, Endoscopic Transpapillary Gallbladder Drainage, Endoscopic Transpapillary Gallbladder Stenting, IYO-stent

## Abstract

**Background and Aims:**

Long-term outcomes of endoscopic transpapillary gallbladder stenting (ETGS) for symptomatic cholelithiasis are unclear. This study aimed to clarify these outcomes of patients with high surgical risks.

**Methods:**

This single-center study included patients with symptomatic cholelithiasis who underwent ETGS between January 2017 and December 2023. Patient background characteristics, endoscopic procedures, stent type used for ETGS, and clinical course were retrospectively assessed. A late adverse event (LAE) was defined as a symptomatic event related to ETGS that occurred more than 2 weeks postoperatively. The frequency of LAEs, LAE-free survival rate, and risk factors for LAEs were analyzed.

**Results:**

A total of 100 patients (median age, 81 years; 57 male and 43 female patients) were included in the analysis. The LAE rate was 27.0% (27/100). LAEs comprised cholangitis (n = 16), solitary cholecystitis (n = 9), solitary liver abscess (n = 1), and cystic artery pseudoaneurysm (n = 1). The estimated 1-year, 2-year, and 3-year LAE-free rates were 88.7%, 80.3%, and 76.7%, respectively, and the estimated median time to LAEs was 5.3 years. Compared with cutoff endoscopic nasobiliary drainage tube placement, IYO-stent placement (hazard ratio, 0.19; 95% confidence interval, 0.07–0.56; *P* < .01) was a significant protective factor against LAE development in the multivariate analysis.

**Conclusion:**

Long-term ETGS with regular follow-up can be safe and effective for patients with high surgical risks and symptomatic cholelithiasis. The use of the IYO-stent was independently associated with a decreased risk of LAEs and potentially contributed to improved long-term LAE-free survival.

## Graphical abstract

**Figure:**
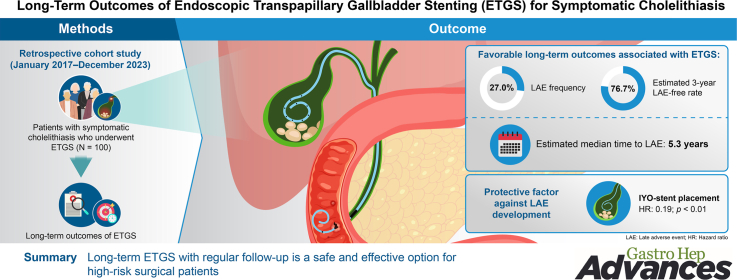

## Introduction

Early or elective cholecystectomy is recommended for patients with gallbladder stones that cause acute cholecystitis and those associated with common bile duct (CBD) stones.^1^ However, because the population is aging, the number of patients with high surgical risks is increasing. Consequently, many cases of cholecystitis and gallbladder stones with or without CBD stones are managed using less invasive treatments such as percutaneous drainage or endoscopic procedures, which often result in gallbladder preservation.

Gallbladder preservation is associated with the risks of cholecystitis and cholangitis recurrence. Cholecystitis recurs in 20.6%–47% of patients treated with conventional percutaneous transhepatic gallbladder drainage (PTGBD) after subsequent tube removal.^2^, ^3^, ^4^ Additionally, CBD stones recur in 14.6% of patients after endoscopic treatment, likely because of migration of residual stones from the preserved gallbladder.^5^ Therefore, preventing disease recurrence related to gallbladder stones in patients undergoing gallbladder-preserving treatments is an important unmet clinical need.

The following two types of endoscopic gallbladder drainage exist: endoscopic transpapillary gallbladder drainage (ETGBD) and endoscopic ultrasound-guided gallbladder drainage (EUS-GBD). EUS-GBD holds increasing promise as a gallbladder drainage method, but it is likely unsuitable for patients with several conditions for which gallbladder wall puncture presents a high risk.^6^ In contrast, ETGBD can be performed even in patients who are avoided for EUS-GBD. ETGBD includes endoscopic transpapillary gallbladder stenting (ETGS) and endoscopic nasogallbladder drainage (ENGBD) and does not require gallbladder wall puncture. The reported technical and clinical success rates of ETGBD are 81%–96% and 75%–97%, respectively.^7^, ^8^, ^9^, ^10^, ^11^, ^12^

ETGS is sometimes permanently placed with the expectation of preventing long-term recurrence of cholecystitis and cholangitis caused by residual stones in the preserved gallbladder. Nevertheless, data regarding the long-term outcomes of ETGS are limited. Therefore, further studies are required to establish its long-term efficacy and safety. Long-term ETGS may be an effective strategy to prevent cholecystitis and cholangitis recurrence in some patients with high surgical risks and gallbladder stones if its long-term efficacy and safety can be validated. Therefore, this study aimed to investigate the long-term outcomes of ETGS.

## Methods

### Patients

This single-center retrospective cohort study was conducted at Nagaoka Red Cross Hospital. Patients who underwent ETGS for biliary diseases between January 2017 and December 2023 were included. The exclusion criteria were as follows: early or elective cholecystectomy; presence of malignant obstruction; short observation period (<100 d); early ETGS removal within a short observation period; and presence of benign biliary stenosis. Patients with symptomatic cholelithiasis who successfully underwent ETGS and were subsequently scheduled to undergo permanent stent placement were analyzed during this study. ETGS or ENGBD was performed as the initial procedure or after symptomatic improvement with PTGBD for acute cholecystitis to enable switching from external biliary drainage to internal biliary drainage. For patients with concomitant CBD stones and gallbladder stones, ETGS was performed following CBD stone removal to prevent further stone migration from the gallbladder to the cystic duct or CBD (Supplementary Figure 1). These approaches were primarily used for patients with high surgical risks. Patients with high surgical risks were those who met at least one of the following criteria: age 80 years or older; American Society of Anesthesiology physical status classification grade ≥3; and age-adjusted Charlson score ≥4.^13^, ^14^, ^15^ ETGS was also performed for patients who declined surgery and opted for ETGS. The demographics and comorbidities of the patients and the results of their endoscopic procedures were retrospectively investigated using the medical records. Data were analyzed in February 2025.

All patients had the opportunity to opt out of the use of their clinical information. This study was approved by the institutional review board of Nagaoka Red Cross Hospital (approval number: 250718) and conducted in accordance with the Declaration of Helsinki.

### Endoscopic Transpapillary Gallbladder Stenting

Prior to ETGS, the initial imaging evaluations were routinely performed using computed tomography and magnetic resonance cholangiopancreatography for many cases; however, for some patients with claustrophobia, a cardiac pacemaker, or a severely poor general condition, magnetic resonance cholangiopancreatography was omitted.

An endoscope (JF-260V or JF-290V; Olympus Medical Systems, Tokyo, Japan) and catheter (PR-V434Q; Olympus) with a 0.025-inch guidewire (Visiglide2; Olympus) were used for endoscopic retrograde cholangiopancreatography (ERCP). Endoscopic sphincterotomy was frequently performed. During biliary stenting, IYO-stent (5-Fr, 32-cm; Gaderius Medical, Tokyo, Japan) or a cutoff endoscopic nasobiliary drainage (ENBD) tube (5-Fr, SilkyPass, pigtail type; Boston Scientific, Natick, MA) was placed in the gallbladder.^16^ IYO-stent placement was usually attempted first. However, when severe cholecystitis or cholangitis required suction and lavage via external drainage, ENBD or ENGBD was performed; subsequently, the nasobiliary drainage tube was cut and left in place for permanent placement. Additionally, a cutoff ENBD tube was also selected in cases where straightening of the cystic duct loop could not be achieved.

### Follow-Up and Definitions of Late Adverse Events

Follow-up examinations (including blood tests, abdominal radiography, and abdominal ultrasonography) were scheduled every 3–6 months after ETGS. In addition to routine outpatient visits, clinical outcomes were assessed when the patients presented outside of their scheduled appointment dates. The last visit date or late adverse event (LAE) onset date was used in the analysis of the observation period. The biliary stent was permanently left in place, and exchange was not planned except in cases of emergency stent occlusion. In accordance with the American Society of Gastrointestinal Endoscopy workshop report, LAEs were defined as symptomatic events that occurred more than 2 weeks after ETGS, and early adverse events were defined as events that occurred during the procedure or within 14 d after ETGS.^17^ When an LAE occurred, treatment was determined based on a discussion between the endoscopist and surgeon and dependent on the condition of the patient at the time.

### Study Outcomes

The primary outcomes of this study were the frequency of LAEs and LAE-free survival rate after ETGS. Secondary outcomes included the results of comparisons between patients with and without LAEs and risk factors for LAE development. Subgroup analyses of late adverse event limited to solitary cholecystitis (LAE-S) and late adverse event limited to cholangitis (LAE-C) were performed because they were the most common types of LAEs and presumed to have distinct underlying causes.

### Statistical Analysis

Categorical variables are expressed as numbers and percentages, and continuous variables are expressed as medians and interquartile ranges (IQRs). Fisher exact test and the Mann–Whitney *U* test were used for categorical and continuous variables, respectively. LAE-free survival was estimated using the Kaplan–Meier method and compared using the log-rank test. Cox proportional hazards models were used to identify risk factors for LAE and calculate the hazard ratio (HR). Variables with *P* < .30 in the univariate analysis were included in the multivariate analysis. Multivariate analyses of the subgroups were not performed because of the small number of patients with LAE-S and LAE-C. Statistical significance was set at *P* < .05. Statistical analyses were performed using SPSS Statistics software (version 26.0; IBM, Armonk, NY) and GraphPad Prism (version 10.0.2; GraphPad Software Inc, La Jolla, CA).

## Results

### Patient Characteristics

**Figure 1:**
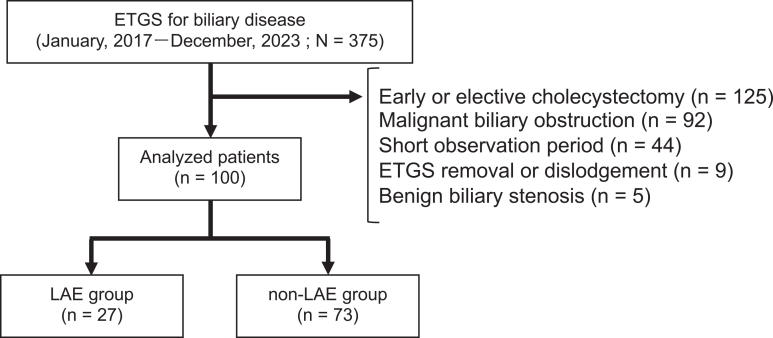
Patient flow in this study. LAE, late adverse event; ETGS, endoscopic transpapillary gallbladder stenting.

A total of 100 patients were included in this study (Figure 1). The patient characteristics and short-term ETGS outcomes are listed in Table 1. The entire cohort had a median age of 81 years (IQR, 71.0–85.3 years) and included 57 male patients and 43 female patients. The median observation period was 1.90 years (IQR, 0.78–3.78 years). Ninety-four of the 100 patients had high surgical risks (94%), and the remaining 6 patients underwent ETGS because they requested it. Patients who used antithrombotic medications accounted for 32% of the sample. Early adverse events were observed in 5 patients (5.0%); of those 5 patients, 4 (4.0%) had mild post-ERCP pancreatitis and 1 (1.0%) experienced bleeding after endoscopic sphincterotomy. No patient experienced perforation of the cholecystic duct. LAEs were observed in 27 patients (27.0%), and the median time from ETGS to LAE occurrence was 1.72 years (IQR, 0.75–3.40 years). No significant differences in patient characteristics of the LAE and non-LAE groups were observed. IYO-stent was used more frequently in the non-LAE group than in the LAE group (93.2% vs 77.8%; *P* = .06, not statistically significantly different). However, in 4 of the 11 patients who received a cutoff ENBD tube, the cystic duct loop could not be straightened, leading to the selection.

**Table 1:** Baseline Characteristics of all Patients and Those of Patients With and Without LAEs

Characteristics	All (N = 100)	Non-LAE group (n = 73)	LAE group (n = 27)	*P* value
Patient characteristics				
Age, y	81 (71.0–85.3)	80 (70.6–85.2)	83 (74.3–85.4)	.70
Sex, male/female	57/43	45/28	12/15	.17
Observation period, y	1.90 (0.78–3.78)	2.24 (0.98–3.98)	1.72 (0.75–3.40)	.32
Patients with high surgical risks	94 (94.0)	68 (93.2)	26 (96.3)	.67
Age ≥80 y	53 (53.0)	38 (52.1)	15 (55.6)	.76
Age-adjusted Charlson score ≥4	92 (92.0)	67 (91.8)	25 (92.6)	.36
ASA-PS ≥3	30 (30.0)	23 (31.5)	7 (25.9)	.49
Antithrombotic medications	32 (32.0)	22 (30.1)	10 (37.0)	.63
Cholecystitis severity				
TG grade I	48 (48.0)	37 (50.7)	11 (40.7)	.09
TG grade II	27 (27.0)	15 (20.5)	12 (44.4)	
TG grade III	6 (6.0)	6 (8.2)	0 (0)	
TG grade II + III	33 (33.0)	21 (28.8)	10 (44.4)	.16
Cholangitis severity				
TG grade I	40 (40.0)	29 (39.7)	11 (40.7)	.35
TG grade II	10 (10.0)	7 (9.6)	3 (11.1)	
TG grade III	8 (8.0)	8 (11.0)	0 (0)	
TG grade II + III	18 (18.0)	15 (20.5)	3 (11.1)	.38
Gallstone pancreatitis	10 (10.0)	7 (9.6)	3 (11.1)	1.00
Gallbladder stones	93 (93.0)	69 (94.5)	24 (88.9)	.38
CBD stones	55 (55.0)	40 (54.8)	15 (55.6)	1.00
ETGS procedure				
Biliary drainage prior to ETGS	51 (51.0)	36 (49.3)	15 (55.6)	.66
Other endoscopic biliary drainage performed simultaneously with ETGS	30 (30.0)	22 (30.10)	8 (29.6)	1.00
EST (including post-EST)	99 (99.0)	72 (98.6)	27 (100)	1.00
IYO-stent placement rate	89 (89.0)	68 (93.2)	21 (77.8)	.06
Early adverse events	5 (5.0)	4 (5.4)	1 (3.7)	.72
Post-ERCP pancreatitis	4 (4.9)	3 (4.1)	1 (3.7)	1.00
Bleeding after EST	1 (1.0)	1 (1.4)	0 (0)	1.00
Others (Perforation of the cholecystic duct, etc.)	0 (0)	0 (0)	0 (0)	1.00

The diagnosis and severity of cholecystitis and cholangitis were judged based on Tokyo Guidelines 2018 (TG 2018).^18^^,^^19^ Severe cholecystitis and cholangitis in this study were defined as grade II + III according to TG 2018.

ASA-PS, American Society of Anesthesiologists physical status; CBD, common bile duct; LAE, late adverse event; EST, endoscopic sphincterotomy; ETGS, endoscopic nasogallbladder drainage.

### Long-Term Endoscopic Transpapillary Gallbladder Stenting Outcomes and Risk Factors for Late Adverse Events

**Figure 2:**
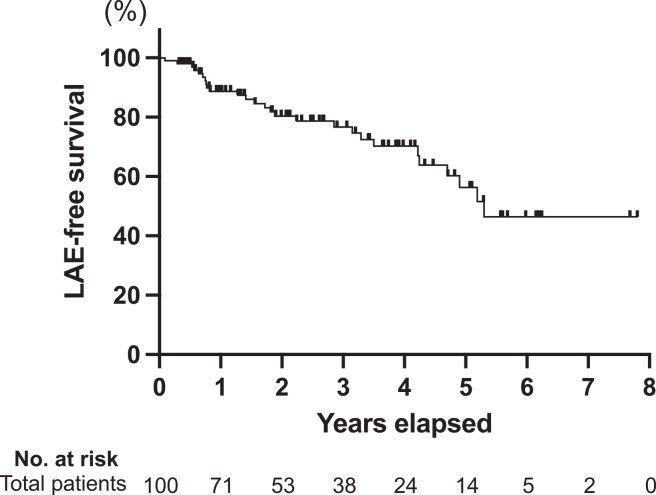
Kaplan–Meier method of determining the LAE-free rate of all patients. LAE, late adverse event.

Using the Kaplan–Meier method, the estimated 1-year, 2-year, and 3-year LAE-free rates were 88.7%, 80.3%, and 76.7%, respectively, and the estimated median time to LAEs was 5.3 years (Figure 2).

A univariate Cox proportional hazards analysis showed that IYO-stent placement was a significant factor that protected against LAE occurrence (HR, 0.26; 95% confidence interval [CI], 0.11 to 0.71; *P* = .01). In the multivariate analysis, IYO-stent placement was also a significant factor that protected against LAE occurrence (HR, 0.19; 95% CI, 0.07–0.56; *P* < .01) (Table 2).

**Table 2:** Cox Proportional Hazards Analysis of Factors Associated With LAE Development

Variables	Univariate		Multivariate	
	HR (95% CI)	*P* value	HR (95% CI)	*P* value
Age, y	1.02 (0.99–1.06)	.25	1.03 (0.99–1.07)	.14
Sex, male/female	0.60 (0.28–1.28)	.19	0.52 (0.23–1.16)	.11
Antithrombotic medications	1.31 (0.60–2.86)	.50		
Severe cholecystitis	1.28 (0.60–2.76)	.52		
Severe cholangitis	0.54 (0.16–1.82)	.32		
Gallstone pancreatitis	1.00 (0.30–3.34)	1.00		
Gallbladder stones	0.28 (0.08–0.99)	.05	0.48 (0.13–1.77)	.27
CBD stones	1.13 (0.53–2.42)	.76		
Biliary drainage prior to ETGS	1.43 (0.67–3.09)	.36		
Other endoscopic biliary drainage performed simultaneously with ETGS	1.01 (0.44–2.31)	.99		
EST (including post-EST)	20.4 (0.00–Inf)	.92		
IYO-stent placement rate	0.26 (0.11–0.71)	.01	0.19 (0.07–0.56)	<.01

CBD, common bile duct; CI, confidence interval; HR, hazard ratio; Inf, infinite; EST, endoscopic sphincterotomy; ETGS, endoscopic transpapillary gallbladder stenting; LAE, late adverse event.

**Figure 3:**
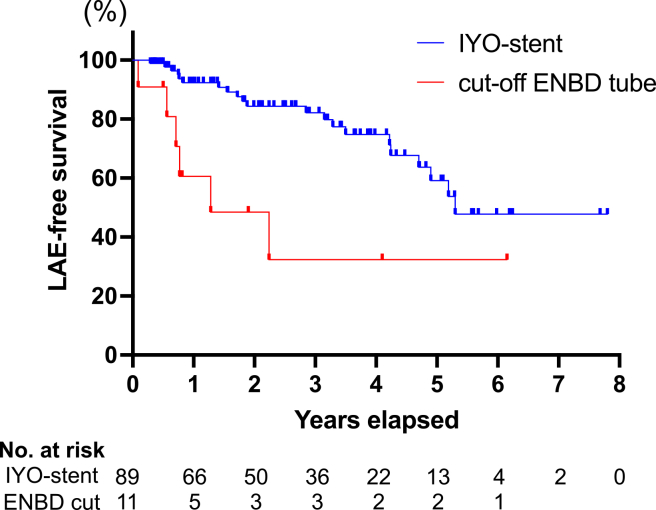
Kaplan–Meier method of determining the LAE-free rates of the IYO-stent group and cutoff ENBD tube group. ENBD, endoscopic nasobiliary drainage; LAE, late adverse event.

Because stent type was a significant factor in the multivariate analysis, we evaluated LAE-free survival stratified by this variable using the Kaplan–Meier method. The LAE-free survival rate associated with IYO-stent placement was significantly improved compared with that associated with cutoff ENBD tube placement (HR, 0.29; 95% CI, 0.07–1.19; *P* < .01) (Figure 3).

**Figure 4:**
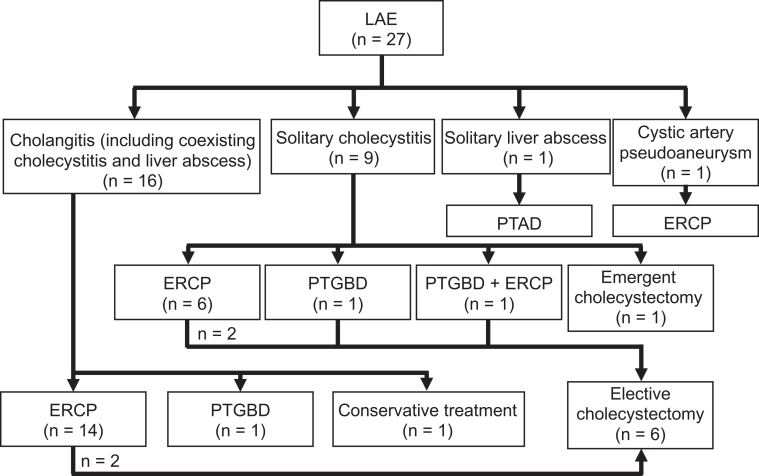
Characteristics of 27 patients with LAEs and their respective treatments. ERCP, endoscopic retrograde cholangiopancreatography; PTAD, percutaneous transhepatic abscess drainage; PTGBD, percutaneous transhepatic gallbladder drainage.

The 27 LAEs comprised cholangitis (n = 16), including concomitant cholecystitis and liver abscess, solitary cholecystitis (n = 9), solitary liver abscess (n = 1), and cystic artery pseudoaneurysm (n = 1), which is a rare LAE. Twenty-one of the LAEs were treated with ERCP alone, 3 were treated with PTGBD or percutaneous transhepatic abscess drainage, 1 was treated with a combination of PTGBD and ERCP, 1 was treated with emergent cholecystectomy, and 1 was treated conservatively because of the poor general condition of the patient who subsequently died. Six patients subsequently underwent elective cholecystectomy (Figure 4).

## Results of Subgroup Analyses

Two subgroup analyses were performed. The results of comparisons between LAE-S and non-LAE and those between LAE-C and non-LAE are shown in Supplementary Tables 1 and 2, respectively. Regarding the results of the comparison between LAE-S and non-LAE, IYO-stent placement was also a significant factor associated with LAE-S in the univariate analysis (HR, 0.19; 95% CI, 0.05–0.74; *P* = .02) (Supplementary Table 3). In contrast, regarding the results of the comparison between LAE-C and non-LAE, the univariate analysis identified the presence of gallbladder stones as a statistically significant factor (HR, 0.10; 95% CI, 0.02–0.44; *P* < .01) (Supplementary Table 4). Additionally, a comparison of the timing of LAE-S and that of LAE-C indicated that LAE-S development (median time to LAE-S, 0.76 years) occurred significantly earlier than LAE-C development (median time to LAE-C, 2.55 years) (*P* < .01).

## Discussion

This study revealed that ETGS was associated with favorable long-term outcomes and an estimated 3-year LAE-free rate of 76.7%. Therefore, long-term ETGS may be an effective treatment option for patients with high surgical risks and symptomatic cholelithiasis. Additionally, the use of IYO-stent significantly reduced the risk of LAE. Moreover, even when LAEs did occur, most patients experienced favorable outcomes with appropriate management. Subgroup analyses further indicated that IYO-stent placement may be particularly beneficial for preventing LAE-S.

A previous study demonstrated that the incidence of LAE associated with permanent ETGS was significantly lower than that associated with temporary drainage using PTGBD or ENBD during a 1-year follow-up period (5.0% vs 22.1%; *P* = .002).^20^ In the present study, we further demonstrated the long-term efficacy and safety of ETGS over a prolonged observation period of approximately 2 years, consistent with a previous study that reported a long-term biliary event-free rate of 76%.^12^ Moreover, a recent study reported the safety and effectiveness of ETGS for patients with gangrenous cholecystitis, which is a severe form of acute cholecystitis.^21^ Collectively, these findings suggest that ETGS is an effective long-term biliary drainage option, even for cases of severe acute cholecystitis in patients with high surgical risks.

Although the long-term outcomes of ETGS were favorable, LAEs could not be completely avoided. Nevertheless, most patients with LAEs in this study were successfully treated with appropriate management. However, 1 older patient with a poor general condition died of cholangitis (grade III according to the Tokyo guidelines III)^18^ despite conservative treatment, underscoring the importance of regular follow-up after ETGS placement. Because a consensus regarding the optimal follow-up examinations and intervals has not yet been determined, we routinely perform blood tests, abdominal radiography, and abdominal ultrasound during each visit to detect asymptomatic recurrent CBD stones and cholestasis. This regular surveillance may contribute to favorable outcomes because it allows early detection and management of LAEs. Patients with severe complications require particularly careful monitoring.

Few studies have investigated risk factors for LAEs after ETGS. One study identified single-stent placement and stent removal as risk factors for LAE-S.^22^ However, double-stent placement, instead of single-stent placement, in ETGS may worsen cholestasis in the CBD, suggesting that LAE-C investigations are indispensable. Furthermore, double-stent placement is not feasible in narrow cystic ducts and cannot be considered a universal method. We identified the use of IYO-stents as a factor that protects against LAEs. One study reported favorable outcomes of acute cholecystitis treated with ETGS using IYO-stents and technical and clinical success rates of more than 90% without recurrence of cholecystitis during a median observation period of 0.85 years^23^; however, that study included a small number of cases (n = 11). Our study is the first to clearly demonstrate that IYO-stents, compared with cutoff ENBD tubes, reduce the risk of LAEs, particularly LAE-S. The IYO-stent (5-Fr, 32-cm) has a unique spiral structure on the gallbladder side, with 36 side halls every 5 mm and a pigtail structure on the duodenal side with 6 side halls, which may result in less frequent stent occlusion compared with that associated with the cutoff ENBD tube. However, in 4 of the 11 patients who received a cutoff ENBD tube, anatomical difficulty related to the cystic duct led to the selection of a cutoff ENBD tube because of concerns regarding potential migration of IYO-stent, which may have introduced selection bias. Additionally, although the present study compared only these 2 types of stents, further studies are warranted to identify the most suitable biliary stent for long-term gallbladder placement, including IYO-stents and other biliary stents.

The presence of gallbladder stones was also a significant factor in the subgroup analysis of LAE-C; however, it is theoretically implausible that the presence of gallbladder stones reduced recurrent cholangitis after ETGS. This finding may have been attributable to the lack of uniformity of imaging studies, which is a limitation of retrospective analyses. Because some patients did not undergo endoscopic ultrasonography or magnetic resonance imaging before ERCP, small stones, biliary debris, and stones not detected using computed tomography may have been overlooked. Studies that include standardized imaging protocols are necessary to reliably assess the presence of stones and clarify their clinical significance.

In recent years, both the American Society of Gastrointestinal Endoscopy and European Society of Gastrointestinal Endoscopy have recommended EUS-GBD over ETGBD for patients with acute cholecystitis who cannot undergo cholecystectomy.^6^^,^^24^ Multiple comparative studies demonstrated that EUS-GBD is associated with higher technical success rates (94%–100%) and clinical success rates (90%–99%) than those associated with ETGBD and revealed the absence of a significant difference in procedure-related adverse events.^7^^,^^25^, ^26^, ^27^, ^28^ However, a network meta-analysis that compared PTGBD, ETGBD, and EUS-GBD showed that disease-specific mortality was lowest with ETGBD and highest with EUS-GBD.^29^ Reports of high technical success rates for EUS-GBD from high-volume expert centers have continued to emerge. However, when introducing EUS-GBD at individual institutions, caution must be paid to the occurrence of intraprocedural adverse events, and dislodgement of a metallic stent can result in fatal outcomes, especially in patients with high surgical risks. Therefore, EUS-GBD should be performed only by endoscopists with substantial expertise in interventional EUS. Additionally, EUS-GBD should be avoided when gallbladder wall puncture is challenging, such as in patients with severe coagulopathy, massive ascites, or malignant gallbladder infiltration. Hence, ETGBD is an irreplaceable treatment option for some patients.

This study had certain limitations. First, because this was a retrospective single-center study, the number of patients in the LAE group was relatively small, and statistical power may have been insufficient. Therefore, to draw definitive conclusions, a large-scale multicenter study—ideally a randomized trial that compares different stent types to exclude selection bias—is necessary. Second, asymptomatic patients with stent dislodgement may have been missed because imaging studies were not uniform at the initial visit and during follow-up. As previously mentioned, the use of standardized imaging would be beneficial. Finally, because many patients with gallbladder stents in place require follow-up, a longer observation period is needed to clarify their final clinical course and determine whether these stents have any impact on survival.

## Conclusion

Most patients who underwent ETGS remained free from LAEs for several years. IYO-stent placement may reduce the risk of LAEs after ETGS. An increase in such cases is expected in an aging society; therefore, long-term ETGS with regular follow-up could be a useful treatment option for patients with high surgical risks and biliary stones.

## Supplementary Materials

Supplementary Materials

Extended PDF
