# Supplementary material for: Long-Term Outcomes of Endoscopic Transpapillary Gallbladder Stenting for Symptomatic Cholelithiasis
Source: Gastro Hep Adv. 2026 Jul 3;5(10):101049. doi: 10.1016/j.gastha.2026.101049 (PMC13472032; doi:10.1016/j.gastha.2026.101049)
Supplement: Supplementary file 1 [file mmc1.pdf]

Supplementary Figure 1: Basic treatment strategies for acute cholecystitis and CBD stones concomitant with gallbladder stones.

CBD, common bile duct; ENGBD, endoscopic nasogallbladder drainage; ETGS, endoscopic transpapillary gallbladder stenting; PTGBD/A, percutaneous transhepatic gallbladder drainage/aspiration.

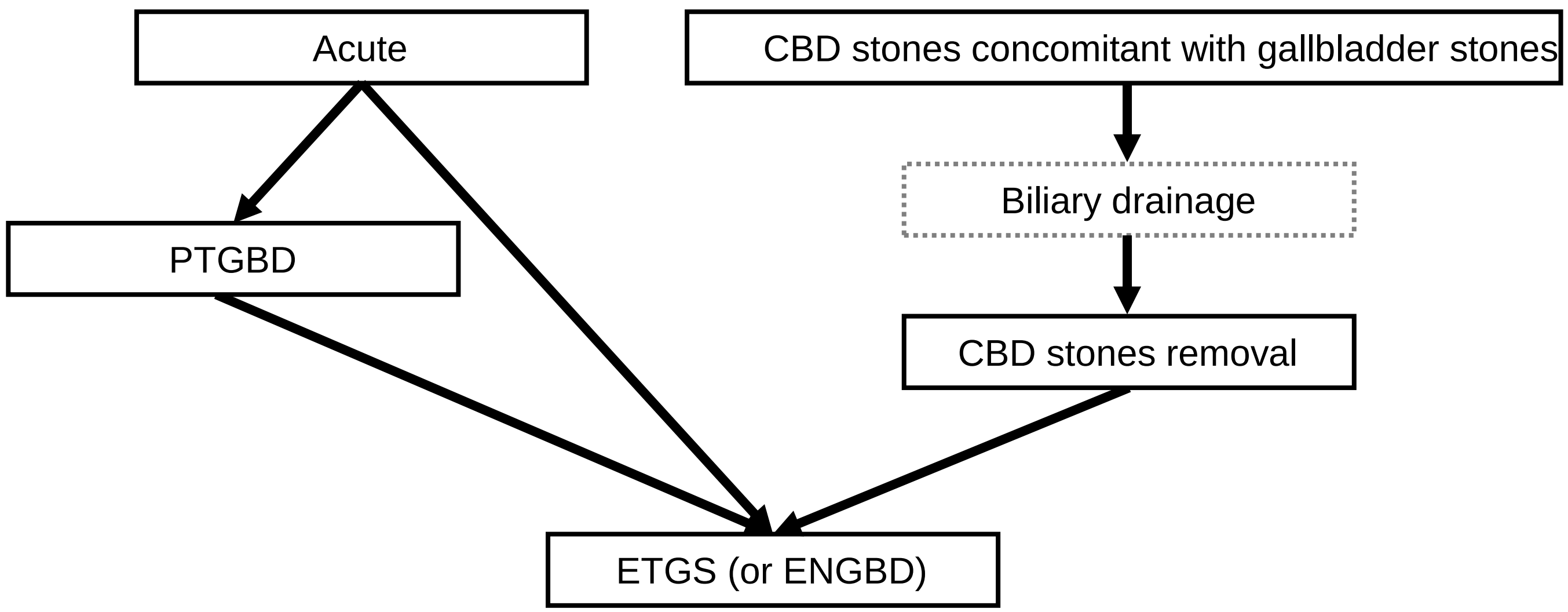

Supplementary Table 1: Baseline characteristics of patients with or without LAE-S

	Non-LAE group (n=73)	LAE-S group (n=9)	*P*-value
Age, years	80 (70.6–85.2)	83 (73.6–84.1)	0.93
Sex, male/female	45/28	5/4	0.73
Observation period, years	2.24 (0.98–3.98)	0.76 (0.53–1.28)	0.01
Antithrombotic medications	22 (30.1)	3 (33.3)	1.00
Severe cholecystitis	21 (28.8)	4 (44.4)	0.45
Severe cholangitis	15 (20.5)	1 (11.1)	0.68
Gallstone pancreatitis	7 (9.6)	0 (0)	1.00
Gallbladder stones	69 (94.5)	8 (88.9)	0.45
CBD stones	40 (54.8)	4 (44.4)	0.73
Biliary drainage prior to ETGS	36 (49.3)	3 (33.3)	0.49
Other endoscopic biliary drainage performed	22 (30.10	3 (33.3)	1.00

simultaneously with ETGS			
EST（including post-EST）	72 (98.6)	9 (100)	1.00
IYO-stent placement rate	68 (93.2)	6 (66.7)	0.04

CBD, common bile duct; EST, endoscopic sphincterotomy; ETGS, endoscopic transpapillary gallbladder stenting; LAE, late adverse event; LAE-S, late adverse event limited to solitary cholecystitis.

Supplementary Table 2: Baseline characteristics of patients with or without LAE-C

	Non-LAE group (n=73)	LAE-C group (n=16)	*P*-value
Age, years	80 (70.6–85.2)	83 (75.2–87.6)	0.28
Sex, male/female	45/28	7/9	0.26
Observation period, years	2.24 (0.98–3.98)	2.55 (1.51–4.22)	0.76
Antithrombotic medications	22 (30.1)	6 (37.5)	0.57
Severe cholecystitis	21 (28.8)	7 (43.8)	0.14
Severe cholangitis	15 (20.5)	2 (12.5)	0.73
Gallstone pancreatitis	7 (9.6)	3 (18.8)	0.66
Gallbladder stones	69 (94.5)	14 (87.5)	0.11
CBD stones	40 (54.8)	9 (56.3)	1.00
Biliary drainage prior to ETGS	36 (49.3)	10 (62.5)	0.41
Other endoscopic biliary drainage performed	22 (30.10	5 (31.3)	1.00

simultaneously with ETGS			
EST (including post-EST)	72 (98.6)	16 (100)	1.00
IYO-stent placement rate	68 (93.2)	14 (87.5)	0.61

CBD, common bile duct; EST, endoscopic sphincterotomy; ETGS, endoscopic transpapillary gallbladder stenting; LAE, late adverse event; LAE-C, late adverse event limited to cholangitis.

Supplementary Table 3: Cox proportional hazards analysis of factors associated with LAE-S development

	Univariate	
	HR (95% CI)	*P*-value
Age, years	1.00 (0.95–1.06)	0.87
Sex, male/female	0.80 (0.21–2.97)	0.74
Antithrombotic medications	1.07 (0.27–4.29)	0.92
Severe cholecystitis	1.61 (0.43–6.00)	0.48
Severe cholangitis	0.58 (0.07–4.62)	0.61
Gallstone pancreatitis	0.04 (0.00–164)	0.56
Gallbladder stones	0.46	0.47

	(0.06–3.69)	
CBD stones	0.69 (0.19–2.58)	0.58
Biliary drainage prior to ETGS	0.57 (0.14–2.27)	0.42
Other endoscopic biliary drainage performed simultaneously with ETGS	1.19 (0.30–4.75)	0.81
EST (including post-EST)	20.46 (0.00–Inf)	0.92
IYO-stent placement rate	0.19 (0.05–0.74)	0.02

CBD, common bile duct; CI, confidence interval; EST, endoscopic sphincterotomy; ETGS, endoscopic transpapillary gallbladder stenting; HR, hazard ratio; LAE, late adverse event; LAE-S, late adverse event limited to solitary cholecystitis.

Supplementary Table 4: Cox proportional hazards analysis of factors associated with LAE-C development

	Univariate	
	HR (95% CI)	*P*-value
Age, years	1.05 (0.99–1.11)	0.09
Sex, male/female	0.51 (0.19–1.36)	0.18
Antithrombotic medications	1.46 (0.53–4.09)	0.46
Severe cholecystitis	1.09 (0.40–2.96)	0.87
Severe cholangitis	0.59 (0.13–2.63)	0.49
Gallstone pancreatitis	1.31 (0.30–5.76)	0.73
Gallbladder stones	0.10	<0.01

	(0.02–0.44)	
CBD stones	1.38 (0.51–3.74)	0.52
Biliary drainage prior to ETGS	1.88 (0.68–5.21)	0.23
Other endoscopic biliary drainage performed simultaneously with ETGS	1.10 (0.38–3.18)	0.86
EST (including post-EST)	Unanalyzable*	Unanalyzable*
IYO-stent placement rate	0.50 (0.11–2.23)	0.37

CBD, common bile duct; CI, confidence interval; EST, endoscopic sphincterotomy; ETGS, endoscopic transpapillary gallbladder stenting; HR, hazard ratio; LAE, late adverse event; LAE-C, late adverse event limited to cholangitis.

* Excluded from the analysis because of the lack of variability (degrees of freedom=0).
